# Patterns of neutralizing humoral response to SARS-CoV-2 infection among hematologic malignancy patients reveal a robust immune response in anti-cancer therapy-naive patients

**DOI:** 10.1038/s41408-022-00608-6

**Published:** 2022-01-18

**Authors:** Cinzia Borgogna, Riccardo Bruna, Gloria Griffante, Licia Martuscelli, Marco De Andrea, Daniela Ferrante, Andrea Patriarca, Abdurraouf Mokhtar Mahmoud, Valentina Gaidano, Monia Marchetti, Davide Rapezzi, Michele Lai, Mauro Pistello, Marco Ladetto, Massimo Massaia, Gianluca Gaidano, Marisa Gariglio

**Affiliations:** 1 Virology Unit, Department of Translational Medicine, University of Piemonte Orientale, Novara, Italy; 2 Division of Hematology, Department of Translational Medicine, University of Piemonte Orientale and “Maggiore della Carità” Hospital, Novara, Italy; 3 CAAD Center for Translational Research on Autoimmune and Allergic Disease, Novara, Italy; 4 Viral Pathogenesis Unit, Department of Public Health and Pediatric Sciences, University of Turin, Turin, Italy; 5 Medical Statistics, Department of Translational Medicine, University of Piemonte Orientale, Novara, Italy; 6 Division of Hematology, University of Piemonte Orientale and “SS Antonio e Biagio e Cesare Arrigo” Hospital, Alessandria, Italy; 7 Division of Hematology, “Santa Croce e Carle di Cuneo” Hospital, Cuneo, Italy; 8 Retrovirus Centre, Department of Translational Medicine and New Technologies in Medicine and Surgery, University of Pisa, Pisa, Italy

**Keywords:** Medical research, Haematological cancer, Infectious diseases

## Abstract

Understanding antibody-based SARS-CoV-2 immunity in hematologic malignancy (HM) patients following infection is crucial to inform vaccination strategies for this highly vulnerable population. This cross-sectional study documents the anti-SARS-CoV-2 humoral response and serum neutralizing activity in 189 HM patients recovering from a PCR-confirmed infection. The overall seroconversion rate was 85.7%, with the lowest values in patients with lymphoid malignancies or undergoing chemotherapy. Therapy-naive patients in the “watch and wait” status were more likely to seroconvert and display increased anti-s IgG titers. Enhanced serum neutralizing activity was observed in the following SARS-CoV-2-infected HM patient groups: *(i)* males; (*ii)* severe COVID-19; and (*iii)* “watch and wait” or “complete/partial response”. The geometric mean (GeoMean) ID50 neutralization titers in patients analyzed before or after 6 months post-infection were 299.1 and 306.3, respectively, indicating that >50% of the patients in either group had a neutralization titer sufficient to provide 50% protection from symptomatic COVID-19. Altogether, our findings suggest that therapy-naive HM patients mount a far more robust immune response to SARS-CoV-2 infection vs. patients receiving anti-cancer treatment, raising the important question as to whether HM patients should be vaccinated before therapy and/or receive vaccine formats capable of better recapitulating the natural infection.

## Introduction

Post-coronavirus disease 2019 (COVID-19) patients with hematologic malignancies (HM) often experience prolonged length-of-hospital stay and high mortality rates—as high as 34%—deriving from their poor general health status and immunosuppressed condition caused by the cancer itself and/or anti-cancer therapies [1–4]. Although HM patients have been prioritized for access to COVID-19 vaccines, emerging evidence indicates that, following vaccination, they are less likely to mount an immune response as robust as that achieved by solid cancer patients or healthy subjects [5–8]. Thus, it is today more urgent than ever to identify the immune correlate(s) of protection (CoPs) from natural SARS-CoV-2 infection that may help predict how HM patients will respond to current COVID-19 vaccines and, at the same time, allow us to understand whether these patients may benefit from alternative vaccine formats that better recapitulate the natural infection.

Neutralizing antibodies (NAbs) are powerful molecules that directly target virus particles and block infection. In addition, they can also eliminate both circulating viruses and infected cells through antibody-mediated effector functions [9]. Thus, NAbs are crucial to overcome infectious diseases and are important CoPs from SARS-CoV-2 re-infection.

While a series of reports have investigated the seroconversion patterns in SARS-CoV-2-infected [10–17] or vaccinated HM patients [18–34], a precise quantification of SARS-CoV-2 neutralizing activity and dynamics, as well as its clinical correlation with antibody response in SARS-CoV-2-infected HM patients, has not been investigated so much in detail yet [35].

The aim of the present study was to determine the humoral response in a cohort of HM patients recovering from documented SARS-CoV-2 infection. To this end, we measured the serum levels of both anti-receptor binding domain of the spike protein (RBD) and anti-spike (s) IgG along with the SARS-CoV-2 neutralizing activity of sera from 189 HM patients and found that therapy-naive patients mount a far better immune response to SARS-CoV-2 infection compared to patients receiving anti-cancer treatment.

## Methods

### Patients, samples, and data collection

This multicenter cross-sectional cohort study involved 3 Hematology Units located in Northern Italy. We included adult patients (aged ≥ 18 years) with any HM and with a laboratory-confirmed SARS-CoV-2 infection identified by reverse transcription polymerase chain reaction (RT-PCR) on nasopharyngeal swab between March 2020 and April 2021 who recovered from COVID-19.

Biological samples were harvested between February 10th and June 10th, 2021. They were provided by UPO Biobank after scientific and ethical review and approval (CE34/21; UPOBB_2021.01_ema-NTA) by the Ethics Committee of Maggiore della Carità Hospital and collaborating hospitals. All participants provided written informed consent; samples and associated data were pseudonymized and recorded on the REDCap (https://www.project-redcap.org/) web application in compliance with current GDPR and Italian legislation on the protection of sensitive data and privacy.

Data on patient characteristics and outcomes were extracted by study investigators from electronic medical records or clinical charts. Patient categories were defined as follows: lymphoid malignancies (Hodgkin and non-Hodgkin lymphoma), myeloid neoplasms (myeloproliferative neoplasm, acute leukemia, myelodysplastic syndrome) or plasma cell disorders (multiple myeloma, solitary plasmacytoma, amyloidosis, monoclonal gammopathy of undetermined significance).

The “watch and wait” status included HM patients who had never been treated but were under active surveillance, whereas patients with a progressive/stable disease displayed an active disease during or after any systemic therapy. In contrast, HM patients with a complete/partial response showed a controlled disease during or after treatment. The “active anti-cancer treatment during SARS-CoV-2 infection” group included SARS-CoV-2-infected HM patients receiving radiation or systemic therapy (both chemotherapy and/or biological agents).

### Quantitative determination of anti-SARS‐CoV‐2 IgG antibodies

A COVID-SeroIndex, Kantaro Quantitative SARS-CoV-2 IgG Antibody RUO Kit (R&D Systems, Bio-Techne, Minneapolis, USA) was employed to detect IgG against the RBD protein [expressed as cutoff index (CI)] and to perform quantitative determination of IgG against the full-length SARS-CoV-2 spike protein [36]. The immunoassay was used and interpreted following the manufacturer’s instructions.

### SARS‐CoV‐2‐specific neutralizing antibody assay

Vero E6 and Vero E6-TMPRSS2 cells—kindly provided by John Hiscott, Pasteur Institute Rome—were cultured in Dulbecco’s modified Eagle’s medium (DMEM) supplemented with 10% fetal calf serum (FCS) (Sigma-Aldrich, Milan, Italy). The replication-competent vesicular stomatitis virus rVSV-eGFP-SARS-CoV-2-S_Δ21_ was a kind gift from Sean P.J. Whelan (Washington University School of Medicine, USA) [37]. The neutralization assay was carried out as previously described [38]. Briefly, diluted sera were incubated with rVSV-SARS-CoV-2-S_Δ21_ at an MOI of 0.05 for 1 h at 37 °C. Ab-virus complexes were added to Vero E6-TMPRSS2 cells in 96-well plates and incubated at 37 °C for 24 h. Subsequently, cells were fixed in 4% formaldehyde (Millipore Sigma) containing DAPI for 15 min on ice. Images were acquired using Operetta (Perkin Elmer) in both the DAPI and GFP channels to visualize nuclei and infected cells (i.e., eGFP-positive cells). Images were segmented using the following building blocks: find nuclei, find cytoplasm, calculate GFP intensity, select population (GFP^+^ cells). Segmentations were then analyzed by counting the GFP^+^ cells out of the number of nuclei, using the Harmony 4.5 software. Acquisitions were further processed to calculate the ID50—the reciprocal dilution inhibiting 50% of the infection—using the Columbus software (Perkin Elmer) (Supplementary Fig. 1).

### Statistical analysis

Data following a non-normal distribution were presented as median and interquartile range (IQR). Categorical variables were summarized as counts and percentages. Differences in medians were evaluated using the Mann–Whitney’s *U*-test. The Kruskal–Wallis test along with the Dunn’s test for multiple comparisons was used to compare more than two groups. The Bonferroni correction method was applied. The Spearman’s correlation coefficient was used to compute correlations between quantitative variables. We examined the association between study variables and Nab levels using both univariable and multivariable logistic regression models. The predictors were incorporated into a multivariable logistic regression model through a stepwise selection process. Odds ratio (OR) and 95% confidence interval (CI) were calculated. A two-sided *p*-value < 0.05 was considered statistically significant. Data were processed using Prism software (GraphPad Prism 9.0) and STATA v.16 (College Station, TX).

## Results

### Seroconversion rate and magnitude of the IgG antibody response to SARS-CoV-2 according to patient clinical characteristics

From March 2020 to April 2021, a total of 189 patients (70 female, 119 male) with a confirmed diagnosis of HM and concomitant SARS-CoV-2 infection were enrolled in this study. The median age at the time of enrollment was 62.6 years (IQR 52–72). They all had RT-PCR-confirmed SARS-CoV-2 infection, and most of the subjects experienced a mild disease (143/189; 75.7%). None of the patients received the vaccine during the study period. Based on HM diagnosis, the patients were grouped into subjects with lymphoid malignancies (*n* = 92; 48.7%), myeloid neoplasms (*n* = 53; 28%), or plasma cell disorders (*n* = 44; 23.3%). Seventy-one patients were receiving anti-cancer treatment when SARS-CoV-2 infection was diagnosed. Circulating IgG Abs against the SARS-CoV-2 spike antigen were measured using the Kantaro Quantitative SARS-CoV-2 IgG Antibody RUO Kit, which allows detecting IgG against both the RBD or the full-length trimeric spike protein of SARS-CoV-2 [36]. The overall percentage of seroconversion in the study cohort was 85.7% (162/189) for both anti-RBD and anti-s IgG, with a positive Spearman’s correlation *r* = 0.85, *p* < 0.0001.

When the patients were stratified according to cancer diagnosis (Table 1), the lowest percentage of seroconversion was found in the lymphoid neoplasm group (75/92; 81.5%), while it reached 88.6% (39/44) and 90.6% (48/53) in the plasma cell and myeloid disorder groups, respectively. Furthermore, patients on chemotherapy-based anti-cancer treatment during SARS-CoV-2 infection displayed a lower seroconversion rate than that of patients receiving a chemotherapy-free regimen (20/27; 74.1% vs*.* 37/44; 84.1%, respectively). Consistent with other reports [25, 30, 32], 5 out of 13 (38.4%) patients who were exposed to B-cell/ plasma cell-depleting monoclonal antibodies during SARS-CoV-2 infection did not seroconvert. Among the 44 patients who had undergone hematopoietic stem cell transplantation (35 autologous, 8 allogeneic, and 1 both), 25% (11/44; 8 autologous and 3 allogeneic) did not seroconvert, and the median years between transplantation and blood sampling was 2.0 (IQR 1.1–3.4) and 4.6 (IQR 2.2–8.4) in seronegative and seropositive patients, respectively (*p* = 0.09). Out of the 8 allogeneic transplantations, 2 patients were under chronic immunosuppression (1 seropositive and 1 seronegative for SARS-CoV-2). In addition, the cancer status at the time of SARS-CoV-2 infection influenced the percentage of seropositivity, which dropped to 69.7 (23/33) in patients with stable/progressive disease, while it rose to 94.9% (35/37) in the “watch and wait” group. Accordingly, univariable logistic regression analysis showed that those patients who seroconverted were more likely to be found in the “watch and wait” (OR = 7.61; 95% CI = 1.53–37.94) or “complete/partial response” group (OR = 2.96; 95% CI = 1.18–7.41).

**Table 1:** Patients characteristics and anti-SARS-CoV-2 seroconversion rate.

	No (%)			
Characteristic	Patients (*n* = 189)	Seropositive (*n* = 162)	Seronegative (*n* = 27)	*p*-value
Age, median (range) years	62.6 (21-87)	62.7 (21-85)	59.8 (33-87)	0.74
Gender				1.00
Male	119 (63.0)	102 (85.7)	17 (14.3)	
Female	70 (37.0)	60 (85.7)	10 (14.3)	
Presence of comorbidities (≥1)	87 (46.0)	73 (83.9)	14 (16.1)	0.51
Cancer diagnosis				0.26
Lymphoid malignancies	92 (48.7)	75 (81.5)	17 (18.5)	
Myeloid neoplasms	53 (28.0)	48 (90.6)	5 (9.4)	
Plasma cell disorders	44 (23.3)	39 (88.6)	5 (11.4)	
Cancer status during SARS-CoV-2 infection				0.01
Watch and wait	37 (19.6)	35 (94.6)	2 (5.4)	
Stable/Progressive disease	33 (17.5)	23 (69.7)	10 (30.3)	
Complete/Partial response	117 (61.9)	102 (87.2)	15 (12.8)	
Active anti-cancer treatment during SARS-CoV-2 infection	71 (37.6)	57 (80.3)	14 (19.7)	0.11
Chemotherapy-based treatment during SARS-CoV-2 infection	27 (38.0)	20 (74.1)	7 (25.9)	0.30
Chemotherapy-free treatment during SARS-CoV-2 infection	44 (62.0)	37 (84.1)	7 (15.9)	
Severe/ Critical COVID-19	45 (23.8)	42 (93.3)	3 (6.7)	0.11
Time from first SARS-CoV-2-positive test to antibody testing ≤ 6 months	152 (80.4)	131 (86.2)	21 (13.8)	0.71
Time from first SARS-CoV-2-positive test to antibody testing > 6 months	37 (19.6)	31 (83.8)	6 (16.2)	

As the timing of blood sampling ranged from 26 to 411 days post SARS-CoV-2 PCR-positive test, we decided to divide the patients into two groups: “<6 months” and “>6 months” since the first SARS-CoV-2 PCR-based-positive test [median 100.5 days (IQR 70.5–132) vs. 295 days (IQR 211–341), respectively]. Using this temporal grouping, we found that the median level of the anti-s IgG titer significantly dropped in the >6-month group [median 80.3 (IQR 23.1–159.7) vs. 29.8 (IQR 18.0–61.2); *p* = 0.002].

**Fig. 1:**
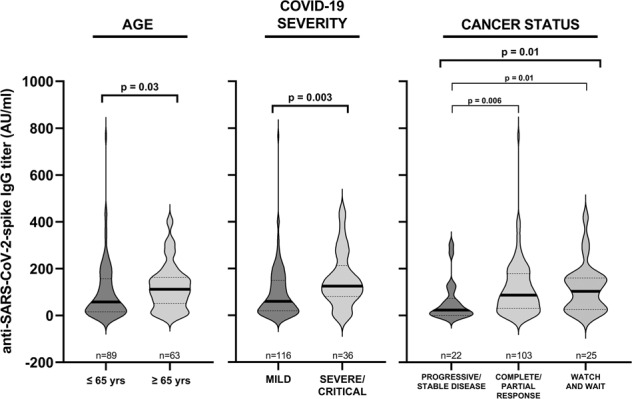
Association of anti-SARS-CoV-2-spike IgG titer with age, COVID-19 severity, and cancer status. Violin plots depicting anti-s IgG titers, measured within 6 months from the first positive SARS-CoV-2 PCR, in hematologic malignancy (HM) patients grouped according to age, COVID-19 severity, and cancer status. Bars represent median (thick line) and interquartile range (dotted line). Statistical analysis was performed by Kruskal–Wallis and Dunn’s tests.

Next, we analyzed how age, gender, comorbidities, cancer diagnosis, COVID-19 severity, cancer status, or active anti-cancer treatment during SARS-CoV-2 infection correlated with the anti-s humoral response. As shown in Fig. 1, we found the median anti-s IgG titer to be significantly higher in older individuals (*p* = 0.03) compared to that of the <65 years group. Furthermore, the levels of anti-s IgG antibodies were higher in individuals with severe/critical COVID-19 (*p* = 0.003) in comparison with those detected in mild disease patients (Fig. 1). Lastly, patients in the “complete/partial response” or “watch and wait” group displayed a significantly higher median anti-s IgG titer compared to that of patients with stable/progressive disease (*p* = 0.006 and *p* = 0.01, respectively).

### Association between clinical factors and anti-SARS-CoV-2 neutralizing activity

**Fig. 2:**
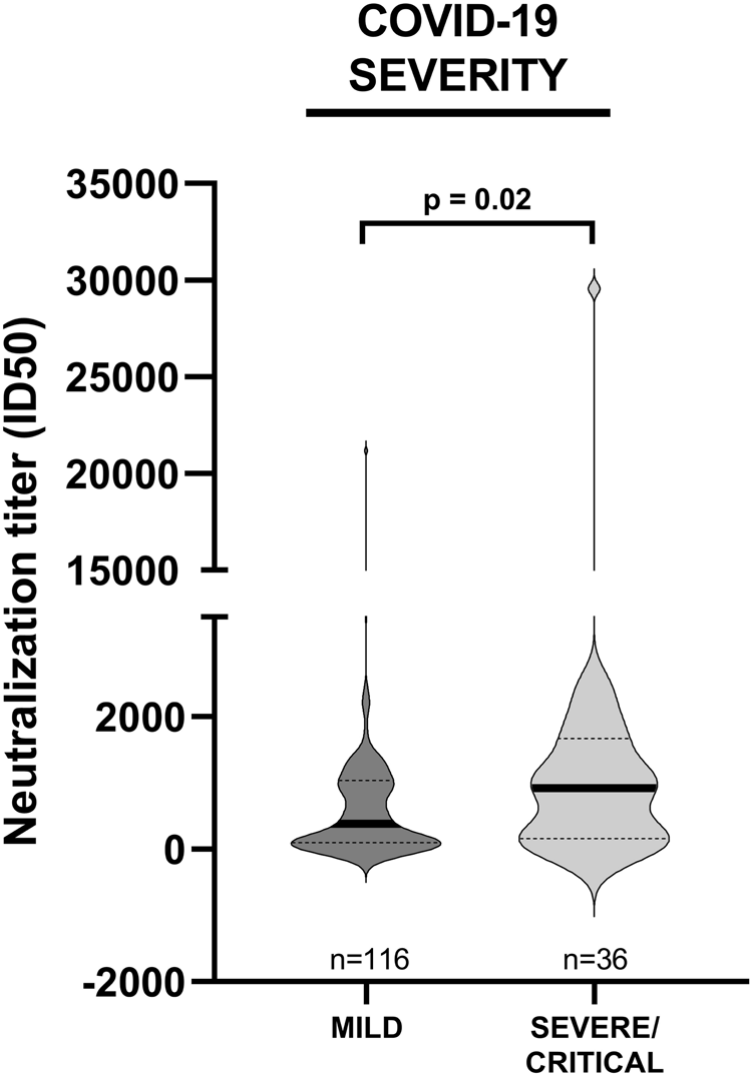
Association of the ID50 neutralization titer with COVID-19 severity. Violin plot depicting the neutralizing activity, measured within 6 months from the first positive SARS-CoV-2 PCR, in HM patients grouped according to COVID-19 severity. Bars represent median (thick line) and interquartile range (dotted line). Statistical analysis was performed by Kruskal–Wallis and Dunn’s tests.

Given that the determination of the neutralizing effects of anti-SARS-CoV-2-spike Abs is critical to understand the protective effects of the immune response, NAb levels were quantified by testing the sera against rVSV-SARS-CoV-2-S_Δ21_ infection of Vero E6-TMPRSS2 cells, as previously described [38]. We observed that the sera with values above the median titer for both anti-s and anti-RBD IgG displayed a significantly higher neutralizing activity than that of sera with values below the median value (*p* < 0.0001 for both comparisons), with a low positive Spearman’s correlation *r* = 0.41, *p* < 0.0001 and *r* = 0.36, *p* < 0.0001, respectively. The geometric mean (GeoMean) ID50 neutralization titer was 299.1 (95% CI = 216.8–412.7) in the <6-month group vs. 306.3 (95% CI = 143.4–654.3) in the >6-month group. We next sought to determine how age, gender, comorbidities, cancer diagnosis, cancer status, active treatment, or COVID-19 severity would correlate with the neutralizing response against SARS-CoV-2 in the <6-month group. The NAb response was significantly higher in patients who experienced severe/critical COVID-19 (median = 955.1, IQR 170.2–1683.5) compared to that in mild disease patients (*p* = 0.02) (Fig. 2). This difference was not evident in >6-month patients, which was probably due to the development of a stabilized neutralizing activity that had flattened the differences in intensity detectable at earlier time points. Using both the univariable or multivariable logistic regression analysis, including age, gender, comorbidities, cancer diagnosis, COVID-19 severity, cancer status, and active anti-cancer treatment during SARS-CoV-2 infection as variables, there was a significant association between gender (OR = 0.5; 95% CI = 0.2–0.9), cancer status—“watch and wait” and “complete/partial response” vs. “stable/progressive disease” during SARS-CoV-2 infection: OR = 2.4; 95% CI = 0.6–9.2 vs. OR = 2.1; 95% CI = 0.7–6.1, respectively—COVID-19 severity (OR = 1.7; 95% CI = 0.7–4.0), and NAb levels (Supplementary Table 1). Males, patients in the groups “watch and wait” or “complete/partial response during SARS-CoV-2 infection”, and patients who experienced severe/critical COVID-19 all displayed enhanced neutralizing activity (Supplementary Table 1).

Next, using the mathematical modeling approach developed by Miles P. Davenport and co-workers, which provides a quantitative prediction of the link between neutralizing antibody levels and clinical protection, we estimated the 50% protective neutralization level against SARS-CoV-2 infection in our cohort to be 256.64 in the <6-month group, calculated as 20.2% of the mean level [39]. Using this predictive model and threshold, we found that 58.6% (89/152) of the subjects in the <6-month group and 51.4% (19/37) in the >6-month group were above this value. Lastly, we stratified patients according to HM type and found that 52.6% (40/76), 66.7% (28/42), and 61.8% (21/34) of patients diagnosed, respectively, with lymphoid malignancies, myeloid neoplasms, or plasma cell disorders displayed (Nab please change in NAb) activities above the 50% protective neutralization level.

## Discussion

To our knowledge, this is the largest cross-sectional study evaluating the anti-SARS-CoV-2 humoral response, alongside neutralizing activity, among SARS-CoV-2-infected HM patients. The observed seroconversion rate in this population, as measured by both anti-RBD and anti-s IgG levels, was 85.7%, which is consistent with previously reported values [10, 16, 35] and lower than that observed in healthy individuals, where it normally ranges from 90 to 100% [40, 41]. Notably, some remarkable differences were found among patient groups. Specifically, we found that patients in the “watch and wait” group had a higher probability to seroconvert upon natural infection compared to patients in the “stable/progressive” and “responsive to therapy” groups, indicating that treatment-naive patients are more likely to efficiently react to SARS-CoV-2 infection.

With regard to the magnitude of the humoral response, HM generally elicited a good humoral response, which was higher in patients in the “watch and wait” status than that observed in patients on treatment regimen. Although the anti-s IgG titers observed in <6-month patients were significantly higher than those in >6-month patients, the median anti-s IgG titers recorded in these two separate groups—80.3 (IQR 23.1–159.7) vs. 29.8 AU/ml (IQR 18.0–61.2), respectively—indicate that any of these patients could mount a humoral response similar to that observed in healthy subjects. Indeed, in a prior study analyzing a cohort of healthy subjects using the same ELISA kit, we found a median anti-s IgG titer of 41.9 and 22.7 AU/ml at 2 and 10 months post SARS-CoV-2 infection [42]. Consistent with many reports performed in healthy subjects, significantly higher anti-s IgG titers were found in older HM patients and in those experiencing severe COVID-19 [43, 44]. Likewise, when we measured the anti-SARS-CoV-2 neutralizing response, remarkably high ID50 titers were found in males, older patients, and those with severe COVID-19, with a slight reduction from <6-month to >6-month patients, which was however not statistically significant [43]. Enhanced neutralizing activity was also significantly associated with the “watch and wait” status. Specifically, 58.6% (<6 months post-infection) and 51.4% (>6 months post-infection) of the patients showed a neutralization titer that was deemed to be sufficient to provide 50% protection from symptomatic COVID-19. Likewise, almost half of the patients in each cancer category (e.g., lymphoid, myeloid, or plasma cell disorders) displayed a neutralizing activity above the cut-off level required for 50% protection. In addition, using the ID50 neutralization titer cut-off range identified by vaccine studies and those reported by studies on high SARS-CoV-2 attack rates showing that the neutralizing activities within a GeoMean range of 1:100-1:200 were strong enough to prevent SARS-CoV-2 re-infection [45, 46], we found that 59.2% (90/152) of the patients in the <6-month group and 51.3% (19/37) of those in the >6-month group displayed ID50 neutralizing titer >200.

Taken together, our findings barely fit with the emerging data obtained in HM patients after mRNA-based COVID-19 vaccination, indicating that HM patients generally show a suboptimal humoral response to vaccination. This impaired response has been reported to be more relevant in some types of HM, mostly chronic lymphocytic leukemia and non-Hodgkin lymphoma, and in patients receiving specific anti-cancer treatment, mostly those targeting B-cell functions [25, 30, 32]. Considering that the exposure of the immune system to viral antigens during natural infection is extremely different from that occurring upon vaccination, it is not totally unexpected to observe a much stronger immune response to naturally infected cells, where the extent of viral antigen exposure is far greater than that triggered by recombinant RNA-based vaccines.

Several shortcomings of our study need to be listed. These include the limited representation of some patient categories, which does not allow us to draw any clear conclusions regarding the differences in the humoral response when the multivariable regression analysis was performed—e.g., small cohorts of patients who received specific anti-cancer therapies known to impair vaccine immunogenicity. Thus, increasing the number of patients may allow the identification of other differences that were not statistically significant in our study. Another limitation is that we did not analyze the levels of SARS-CoV-2-specific T-cell responses. Further analyses are clearly needed to correlate virus neutralization with cellular immunity and to assess their longitudinal dynamics in these patients. Finally, as the study design only included COVID-19 HM patients who recovered from the disease, the rate of SARS-CoV-2 seroconversion is very high, thus limiting the comparison with those who did not seroconvert.

In summary, although our findings confirm the reduced seroconversion in HM patients, especially those with lymphoid disorders or undergoing chemotherapy-based treatment during SARS-CoV-2 infection, the overall neutralizing humoral response upon natural SARS-CoV-2 infection seems to be quite efficient and sustained overtime, with a consistently more robust response in “watch and wait” patients.

Thus, our results raise two important questions whose answers await further research: *(i)* Does the reduced immunogenicity of the mRNA-based COVID-19 being observed in HM patients reflect the immune response elicited by the natural infection?; and *(ii)* Can HM patients benefit from different vaccine formats capable of better mimicking the natural infection?

Overall, our findings suggest that more efforts should be put in place in order to develop tailored vaccine approaches, e.g., boosters or heterologous vaccination, to thoroughly assess the humoral response, and to ensure the effectiveness of SARS-CoV-2 vaccination in this unique population of patients.

## Supplementary information

Supplementary Table 1

Supplementary Figure 1

Supplementary Figure 1 legend
