# Supplementary material for: Patterns of neutralizing humoral response to SARS-CoV-2 infection among hematologic malignancy patients reveal a robust immune response in anti-cancer therapy-naive patients
Source: Blood Cancer J. 2022 Jan 18;12(1):8. doi: 10.1038/s41408-022-00608-6 (PMC8764505; doi:10.1038/s41408-022-00608-6)
Supplement: Supplementary file 1 — Supplementary Table 1 [file 41408_2022_608_MOESM1_ESM.docx]

|  | **Univariable model** | | **Multivariable model** | |
| --- | --- | --- | --- | --- |
|  | **OR** | **95% CI** | **OR** | **95% CI** |
| **Age** |  |  |  |  |
| <65 years | 1 |  | 1 |  |
| ≥65 years | 1.63 | 0.85-3.13 | 1.70 | 0.79-3.65 |
| **Gender** |  |  |  |  |
| Male | 1 |  | 1 |  |
| Female | 0.50 | 0.25-0.98 | 0.46 | 0.23-0.95 |
| **Presence of comorbidities** |  |  |  |  |
| No | 1 |  | 1 |  |
| Yes | 1.17 | 0.62-2.22 | 0.96 | 0.46-1.99 |
| **Cancer diagnosis** |  |  |  |  |
| Lymphoid malignancies | 1 |  | 1 |  |
| Myeloid neoplasms | 1.35 | 0.63-2.87 | 1.54 | 0.65-3.69 |
| Plasma cell disorders | 1.11 | 0.49-2.50 | 1.05 | 0.40-2.78 |
| **Cancer status during SARS-CoV-2 infection** |  |  |  |  |
| Stable/progressive disease | 1 |  | 1 |  |
| Watch and wait | 2.23 | 0.69-7.20 | 2.38 | 0.61-9.24 |
| Complete/partial response | 1.78 | 0.69-4.62 | 2.10 | 0.72-6.10 |
| **Active anti-cancer treatment during SARS-CoV-2 infection** |  |  |  |  |
| No | 1 |  | 1 |  |
| Yes | 1 | 0.51-1.93 | 0.93 | 0.39-2.21 |
| **COVID-19 severity** |  |  |  |  |
| Mild | 1 |  | 1 |  |
| Severe/ Critical | 1.80 | 0.84-3.87 | 1.68 | 0.70-4.01 |

**Supplementary Table 1. Univariable and multivariable regression analysis of the association between patient characteristics and anti-SARS-CoV-2 neutralizing activity (≤ median *vs* > median) in the < 6** **months group.**
