# Supplementary figures and images for: Patterns of neutralizing humoral response to SARS-CoV-2 infection among hematologic malignancy patients reveal a robust immune response in anti-cancer therapy-naive patients
Source: Blood Cancer J. 2022 Jan 18;12(1):8. doi: 10.1038/s41408-022-00608-6 (PMC8764505; doi:10.1038/s41408-022-00608-6)

**a**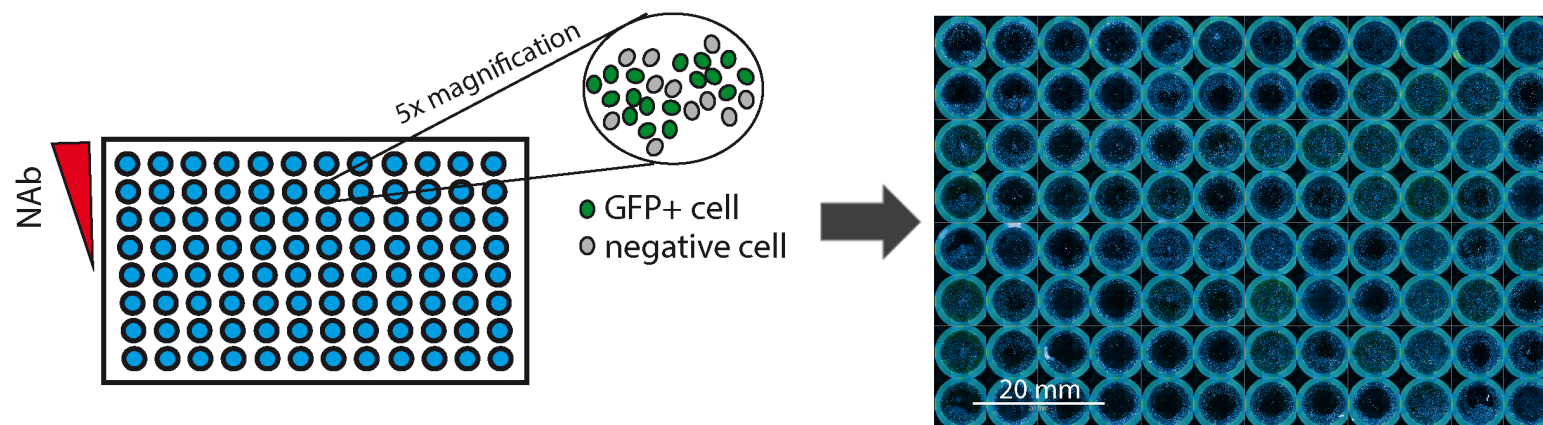**b**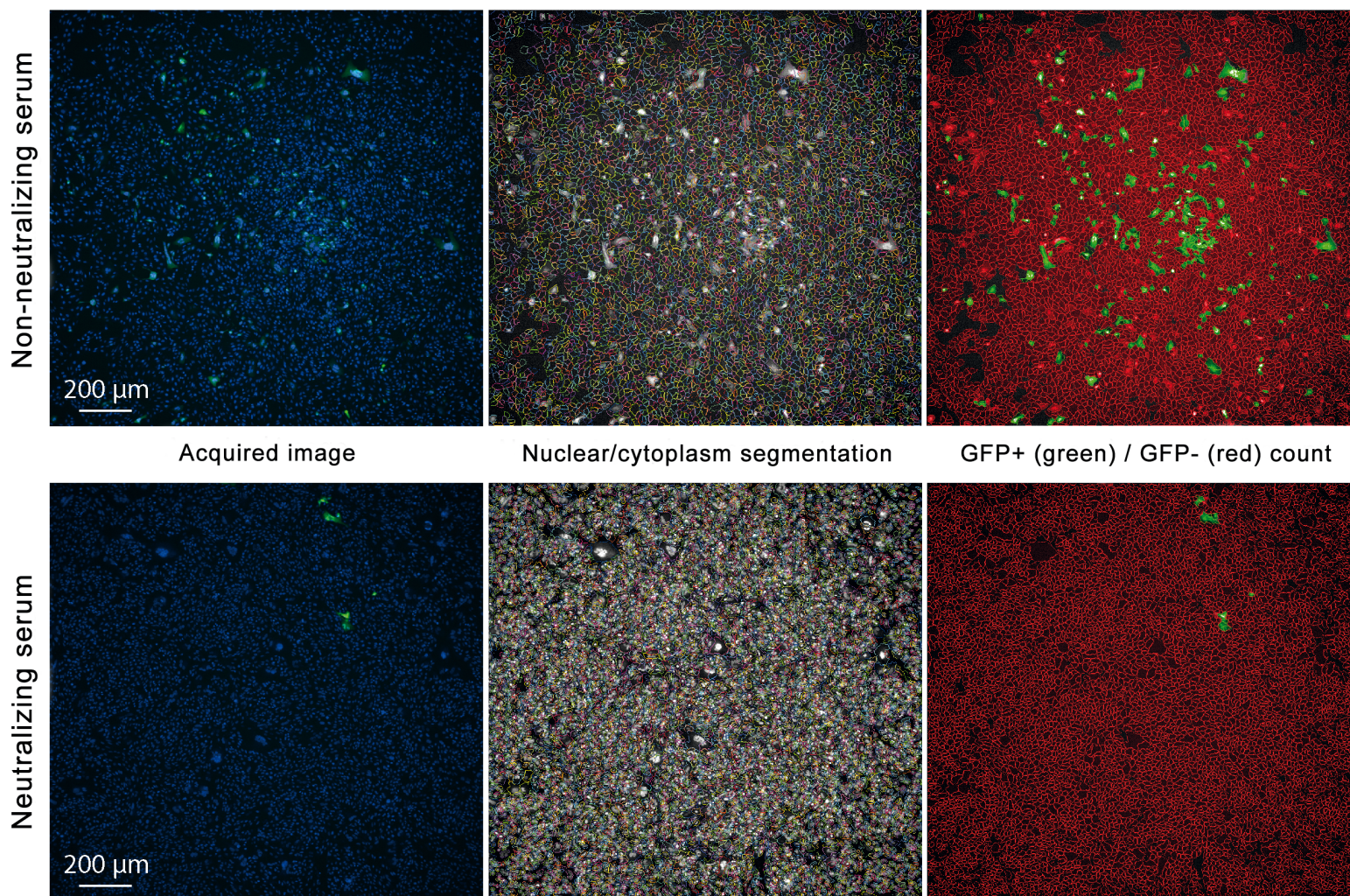

Supplement: Supplementary file 2 — Supplementary Figure 1 [file 41408_2022_608_MOESM2_ESM.pdf]
