# Supplementary material for: Patterns of neutralizing humoral response to SARS-CoV-2 infection among hematologic malignancy patients reveal a robust immune response in anti-cancer therapy-naive patients
Source: Blood Cancer J. 2022 Jan 18;12(1):8. doi: 10.1038/s41408-022-00608-6 (PMC8764505; doi:10.1038/s41408-022-00608-6)
Supplement: Supplementary file 3 — Supplementary Figure 1 legend [file 41408_2022_608_MOESM3_ESM.docx]

**Supplementary Figure 1. SARS‐CoV‐2‐specific neutralizing antibody assay.** Panel**a**shows a schematic diagram of the assay used to assess the anti-SARS-CoV-2 neutralizing activity of patients' sera. Panel **b**shows representative images of the neutralization assay with pictures obtained with a non-neutralizing serum (upper row) or a neutralizing serum (bottom row*).* Images were acquired using Operetta in both the DAPI (blue) and GFP (green) channels, segmented (nuclei, cytoplasm, GFP^+^), and analyzed by counting the GFP^+^ cells out of the number of nuclei, using the Harmony 4.5 software.
